# Time course of VCAM-1 expression in reperfused myocardial infarction in swine and its relation to retention of intracoronary administered bone marrow-derived mononuclear cells

**DOI:** 10.1371/journal.pone.0178779

**Published:** 2017-06-19

**Authors:** André Uitterdijk, Bianca C. W. Groenendijk, Charlotte Gorsse-Bakker, Anna Panasewicz, Stefan Sneep, Dennie Tempel, Esther H. van de Kamp, Daphne Merkus, Willem J. van der Giessen, Dirk J. Duncker

**Affiliations:** Department of Cardiology, Erasmus MC, Rotterdam, The Netherlands; University of Louisville, UNITED STATES

## Abstract

**Background:**

Intracoronary infusion of autologous bone marrow-derived mononuclear cells (BMMNC), after acute myocardial infarction (AMI), has been shown to improve myocardial function. However, therapeutic efficacy is limited, possibly because cell retention rates are low, suggesting that optimization of cell retention might increase therapeutic efficacy. Since retention of injected BMMNC is observed only within infarcted, but not remote, myocardium, we hypothesized that adhesion molecules on activated endothelium following reperfusion are essential. Consequently, we investigated the role of vascular cell adhesion molecule 1 (VCAM-1) in BMMNC retention in swine undergoing reperfused AMI produced by 120 min of percutaneous left circumflex coronary occlusion.

**Methods and results:**

VCAM-1 expression in the infarct and remote region was quantified at 1, 3, 7, 14, and 35 days, post-reperfusion (n≥6 swine per group). Since expression levels were significantly higher at 3 days (2.41±0.62%) than at 7 days (0.98±0.28%; p<0.05), we compared the degree of cell retention at those time points in a follow-up study, in which an average of 43·10^6^ autologous BMMNCs were infused intracoronary at 3, or 7 days, post-reperfusion (n = 6 swine per group) and retention was histologically quantified one hour after intracoronary infusion of autologous BMMNCs. Although VCAM-1 expression correlated with retention of BMMNC within each time point, overall BMMNC retention was similar at day 3 and day 7 (2.3±1.3% vs. 3.1±1.4%, p = 0.72). This was not due to the composition of infused bone marrow cell fractions (analyzed with flow cytometry; n = 5 per group), as cell composition of the infused BMMNC fractions was similar.

**Conclusion:**

These findings suggest that VCAM-1 expression influences to a small degree, but is not the principal determinant of, BMMNC retention.

## Introduction

Cell therapy with autologous bone marrow-derived cells generally yields statistically significant, but rather modest, improvements in myocardial function after acute myocardial infarction (AMI) [1–3]. With 20·10^6^ cardiomyocytes per gram of jeopardized myocardium [4], potentially lost to infarction, it is evident that the absolute number of cells retained to regionally treat the affected area is of great importance. However, cell retention after intracoronary cell therapy is very low, varying widely between studies, possibly as a result of differences in cell type, timing of administration and initial cell dose [5–20]. Previous work from our laboratory showed that cell retention after intracoronary injection of bone marrow-derived mononuclear cells (BMMNCs) at one week of reperfusion in a swine model of AMI, amounted 8% and 6.5%, respectively, at 1.5 hours and 4 days post-injection [14]. Retention of cells, as measured with immunofluorescence, was observed only within the infarcted region, whereas no cells were retained when cells were injected selectively into the non-occluded left anterior descending coronary artery (LAD). The latter findings suggest that cell adherence and retention are active processes, occurring exclusively in the reperfused infarct-zone, and not just physical entrapment of the cells due to cell size.

Following AMI, activated endothelium within the infarct region drives the expression of transmembrane adhesion molecules that mediate leukocyte-endothelium interactions to orchestrate regional immune responses [21, 22]. These damage-associated adhesion molecules serve as primary “loading-docks” for cell anchorage and their limited and transient post-AMI presence may be correlated to the limited retention of infused cells. A key player associated with endothelial adhesion of circulating immune cells is Vascular Cell Adhesion Molecule 1 (VCAM-1) [23]. It is however, largely unknown to what extent VCAM-1 is present in the days-weeks following AMI and to what extent VCAM-1 expression influences BMMNC retention.

In light of these considerations, we investigated *i)* the temporal expression of VCAM-1 in infarcted and remote myocardial regions in swine with reperfused AMI; *ii)*, the correlation of VCAM-1 presence to autologous bone marrow-derived cell retention and *iii)* temporal changes in AMI-induced changes in the composition of the injected BMMNCs.

## Material and methods

### VCAM-1 expression after acute myocardial infarction

Animal experiments were performed in 48, 5–6 month old Yorkshire x Landrace swine of either sex (31.0±0.3kg). All experiments were performed in strict compliance with the “Guide for the Care and use of Laboratory Animals” and were specifically approved by the Animal Ethics Committee of the Erasmus MC Rotterdam, The Netherlands (approval numbers: EUR1871, EMCnr.109-09-12 and EUR2058, EMCnr.109-10-05). All experiments were performed with appropriate and local Animal Ethics Committee approved analgesics, anesthetics and euthanasics (see text below for details) and all efforts were made to minimize any discomfort. Humane endpoints were carefully respected in collaboration with a dedicated and experienced veterinarian. Humane endpoints were defined as premature killing of animals following *i)* severe and permanent behavioral changes including apathy and lethargy or when the animal ceased normal food and water intake. *ii)* Severe cardiorespiratory disease such as acute heart failure with peripheral cyanosis. Or *iii)*, rapid and excessive weight loss (>20% body weight reduction).

#### Surgery

Myocardial infarction was produced in 33 swine (30.5±0.3kg) as previously described [14, 24, 25]. For this purpose, swine were sedated with an intramuscular injection of midazolam (1mg/kg), ketamine (20 mg/kg) and atropine (1mg). Then, an intravenous (iv) ear catheter was placed for induction of anesthesia with thiopenthal sodium (17 mg/kg). Next, animals were intubated and mechanically ventilated (O_2_:N_2_ 1:3 v/v), while anesthesia was maintained with fentanyl (20μg/kg/h iv). Under sterile conditions, a 9F arterial sheath was placed in a dissected carotid artery and anticoagulation was ascertained by the iv administration of 10,000 units of heparin + 5,000 units every additional hour of surgery. Physiological body core temperature was maintained with heating pads [26]. Saline was infused at 100 ml/h iv to maintain fluid status of the animals, while arterial blood pressure and ECG were monitored continuously. The left circumflex coronary artery (LCx) was catheterized under fluoroscopic guidance with a 7F guiding catheter and maximal coronary artery dilation was produced with 1mg isosorbidatedinitrate for optimized balloon sizing. Next, the LCx was visualized with selective infusion of the contrast agent iodixanol and coronary diameter was measured with dedicated software (CAAS, Pie Medical, Eindhoven, The Netherlands). After the selection of the occlusion site by at least two researchers to ascertain optimal protocol adherence, the LCx was occluded for 2h distally to the first marginal branch followed by reperfusion with a standard guide wire and an appropriately sized percutaneous transluminal coronary angioplasty balloon. Following occlusion, anesthesia was switched to isoflurane inhalation anesthesia (1–3% v/v) [14, 27]. After 2h of occlusion the balloon was deflated and reperfusion was allowed. Anesthetized animals were monitored until hemodynamically stable. Antibiotic prophylaxis was given intramuscularly (procainebenzylpenicilline 20 mg/kg and dihydrostreptomycine sulphate 25 mg/kg). Catheters were removed, the incision site was closed and animals were allowed to recover.

#### Follow-up

After 1 (n = 6), 3 (n = 6), 7 (n = 7), 14 (n = 6) or 35 (n = 6) days post-AMI, animals were sedated as described above. Anesthesia was induced (15 mg iv) and maintained with pentobarbital sodium (15 mg/kg/h iv). Following sternotomy, the pericardium was opened and the heart was electrically induced to fibrillate. Next, the heart was excised and rinsed with ice-cold saline. The left ventricle was isolated and cut into transverse sections and both remote and infarct tissue were preserved in optimal cutting temperature compound (Tissue-Tek, Sakura Finetek, Alphen aan den Rijn, The Netherlands) using frozen CO_2_ (dry ice) for subsequent histopathological analyses.

#### Immunohistochemistry

Cryosections of 5μm were fixed in ice-cold acetone for 10 min. Endogenous peroxidase activity was blocked with 0.3% H_2_O_2_ in 40% methanol for 60 min. Adjacent sections were incubated with anti-VCAM-1 (mouse-anti pig, 1:300, gift from Prof. D. Haskard, London, United Kingdom) overnight at 4°C. Next, using the Vectastain biotinylated horse-anti mouse kit (Brunschwig Chemie, Amsterdam, The Netherlands) and diaminobenzidine (DAKO, Eindhoven, The Netherlands) expression was visualized. Stained sections were photographed with a virtual microscope (Hamamatsu NanoZoomer, 2.0-HT Slide Scanner). Whole sections, containing 5–6 high-power fields (40x) were analysed for VCAM-1 presence with dedicated software using a color threshold (BioPix iQ, 2.2.1, BioPix AB, Göteborg, Sweden). Data were expressed as a percentage of the total surface area.

#### RT-PCR

Cryopreserved infarct and remote tissue was homogenized (n = 5 or 6 per group) and RNA was isolated using the RNeasy Fibrous Tissue Mini Kit (Qiagen, Hilden, Germany) according to manufacturer’s instructions. Quantity and Quality (A260/A280 ratio) of isolated RNA was determined with a NanoDrop ND10000 spectrophotometer (Thermo Fischer Scientific, USA) and RNA integrity was confirmed by using the Agilent Bioanalyzer 2100. Next, cDNA was synthesized from 500ng of RNA using the SentiFAST cDNA synthesis kit (Bioline, Luckenwalde, Germany).

Using the comparative Ct (ΔCt) method (forward primer: TGTGAAGGGATTAACCAGGCT, reverse primer: CAGTGTCCCCTTCCTTGACG) VCAM-1 expression was determined. Results are expressed as fold-increase to the normalized day 1 post-AMI remote expression of the housekeeping gene glyceraldehyde-3-phosphate dehydrogenase (GAPDH, forward primer: GCTCATTTCCTCGTACGACAAT, reverse primer: GAGGGCCTCTCTCCTCCTCGC) using the CFX manager software (Bio-Rad). PCR product accurateness was confirmed by sequencing.

### Effects of VCAM-1 expression on cell retention

Based on the results obtained in the VCAM-1 expression studies described above, two time points were selected to test whether regional up-regulation of VCAM-1 leads to increased cell retention after AMI.

#### Surgery, cell isolation and -infusion

In 15 swine (32.3±0.6kg), reperfused AMI was induced as above and serial blood for biomarker measurements was taken as described before [24]. At 3 or 7 days post-AMI (n = 6 surviving pigs per group), bone marrow was harvested under sterile conditions from the ileac crest and/or the proximal femur of the anesthetized pigs as described before [14]. In brief, up to 160 ml bone marrow was aspirated using 5ml heparinized syringes and received in 50 ml centrifuge tubes containing 10 ml phosphate buffered saline (PBS) and 5,000 units of heparin. Bone marrow was selectively enriched for the mononuclear fraction by density centrifugation (20 min at 800g at RT), using equal amounts of Lymphoprep as a separation medium (Lucron, Milsbeek, The Netherlands). Using a sterile pipette, the mononuclear cell fraction was carefully aspirated and filtered using a 100 μl cell strainer. The fraction was washed twice by centrifugation in wash buffer (PBS containing 0.1% of autologous serum, 10 min at 600 g at RT). The obtained cells were resuspended in wash buffer and added to a red blood cell lysis solution for 10 min at RT (1:3, 8.3 g NH_4_Cl + 1.0 g KHCO_3_ + 1.8 ml 5% EDTA in 1000 ml H_2_0) to remove erythrocytes. Enriched and washed cells were collected by centrifugation (2 min at 2000 g at RT). A conventional Bürker-Türk haemocytometer and trypan blue exclusion were used to count total cell number and ascertain viability. Up to 50·10^6^ cells were labeled with the non-cytotoxic fluorescent membrane marker PKH26 (Sigma-Aldrich, Zwijndrecht, The Netherlands) and successful labeling was unremittingly ascertained with fluorescence microscopy of a small aliquot (labelling efficiency >99%; data not shown; see supplemental S1 Fig for a typical example). Labeled cells were then cautiously resuspended in washing buffer to obtain a density of 1.7·10^6^ cells/ml and infused intracoronary using a multi-purpose infusion catheter into the infarcted area of the heart at a rate of 1·10^6^ (slow, n = 2 per group) or 5·10^6^ (fast, n = 4 per group) labeled cells per minute. One hour after the completion of the infusion protocol, the hearts of the deeply anesthetized animals were electrically fibrillated and subsequently excised, and the complete infarct region was subsequently carefully sectioned into 1cm^2^-sized cubes and processed for histopathology as described above.

#### Quantification of retained cells

VCAM-1 was quantified in cell infusion-treated animals as described above. Next, using a checkerboard-like approach, 5 μm cryosections from the infarcts were fixed with ice-cold acetone for 10 minutes. Sections were washed with PBS and mounted with 4',6-diamidino-2-phenylindole (Vectashield with DAPI, Brunschwig Chemie). Using a Zeiss Axiovert S100, 10x photos were taken of the stained sections, converted to grayscale to allow for further software-based analyses and using a color threshold PKH26 positive cells were counted with ImageJ (version 1.46r, National Institutes of Health, USA). Using section thickness, average cell thickness and tissue dimensions we calculated absolute and relative cell retention.

### Effects of myocardial infarction on composition of the mononuclear fraction

#### Flow cytometry

Parallel to processing of cells for intracoronary cell injection experiments, a representative aliquot from each bone marrow aspirate (n = 5 per group, due to technical failure of the flow cytometer during one experiment in each group) was processed for flow cytometry to assess relative contribution of the various cell types within the mononuclear fraction, in order to determine the composition of the mononuclear fraction at 3 vs 7 days post-AMI. For this purpose, we quantified the percentage of B-cells (CD79a^+^, AbD Serotec, Puchheim, Germany), T-cells (CD3^+^, Abcam, Cambridge, UK), α4-integrin positive cells (CD49d^+^, AbD Serotec), β2 integrin positive cells (CD18^+^, VMRD, Pullman, WA, USA), CD34 positive cells (CD34^+^, R&D Systems, Abingdon, UK) and mesenchymal stem cells (as defined by CD105^+^/CD90^+^/CD14^-^/CD45^-^, CD105: Exbio, Prague, Czech Republic; CD90: BD Biosciences, Breda, The Netherlands; CD14 and CD45 AbD Serotec). For the B- and T-cell staining, cells were resuspended in azide/serum/protein-free PBS at a concentration of 10∙10^6^ cells/ml. Fixable Viability Dye, for cell viability selection, was added and incubated for 30 minutes at 4°C. After washing with FACS Flow (BD Biosciences), CD3 antibody was incubated for 15 minutes at room temperature. Cells were washed and incubated with Leucoperm Reagent A for 15 minutes at RT followed by washing and incubation with Leucoperm Reagent B for 30 minutes at RT, final washing and resuspension in FACS Flow. For CD18, CD34, CD49d and their combinations with CD14^-^ and CD45^-^ staining, cells were resuspended in FACS Flow and incubated with the first primary antibodies (CD18, CD49d or CD34) for 15 minutes at room temperature. After washing, cells were incubated with secondary antibodies for 30 minutes at room temperature, followed by washing and incubation with the second set of primary antibodies (CD14 and CD45) for 15 minutes at room temperature. For cell viability selection 7-amino-actinomycin D (7-AAD, BD Biosciences) was added and cells were washed and resuspended in FACS Flow. For the MSC analysis, cells were resuspended in FACS Flow and incubated with the primary antibodies (CD105, CD90, CD14 and CD45) for 15 minutes at room temperature followed by addition of 7-AAD. After washing, cells were resuspended in FACS Flow. Flow cytometric analysis was performed on a FACSCanto (BD Biosciences) and subsequent data analysis by use of FlowJo software (Tree Star Inc, Ashland, OR, USA).

### Statistics

**Fig 1:**
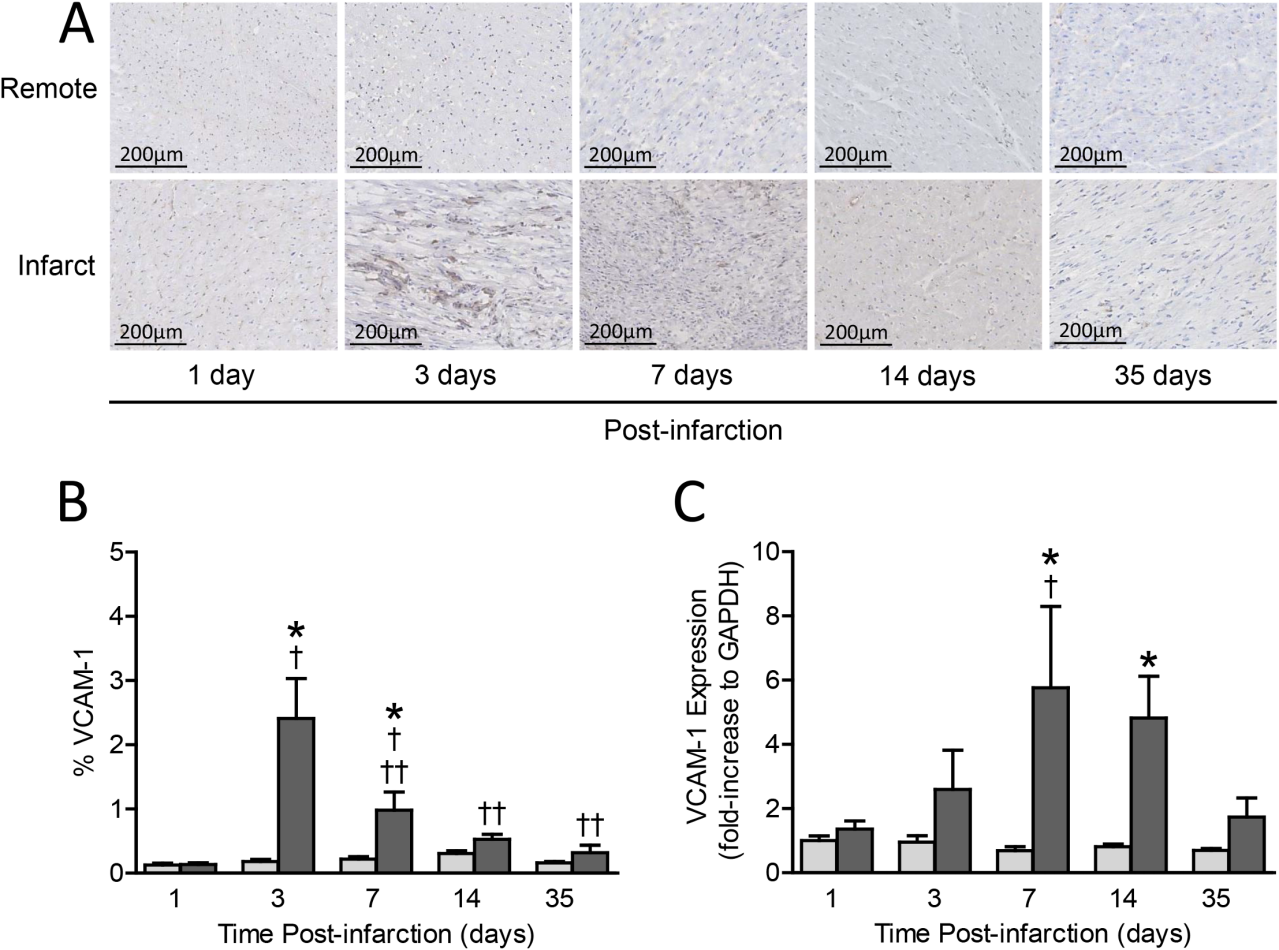
**Panel A**. Typical examples of VCAM-1 presence in infarct and remote myocardial tissue at 1, 3, 7, 14 and 35 days after myocardial infarction. Brown is VCAM-1. **Panel B.** Temporal VCAM-1 presence at 1 (n = 4), 3 (n = 4), 7 (n = 5), 14 (n = 6) and 35 days (n = 6) after myocardial infarction. Data are expressed as mean ± SEM. light grey bar = remote tissue, dark grey bar = infarct tissue. * = p<0.05 vs. corresponding remote; † = p<0.05 vs. day 1; †† = p<0.05 vs. day 3. **Panel C.** Temporal VCAM-1 expression at 1 (n = 6), 3 (n = 5), 7 (n = 6), 14 (n = 6) and 35 days (n = 5) after myocardial infarction. Data are presented as fold-change ± SEM from the VCAM-1 expression in the remote zone on day 1 post-infarct, and corrected for expression of the housekeeping gene glyceraldehyde-3-phosphate dehydrogenase (GAPDH). light grey bar = remote tissue, dark grey bar = infarct tissue. * = p<0.05 vs. corresponding remote; † = p<0.05 vs. day 1.

**Fig 2:**
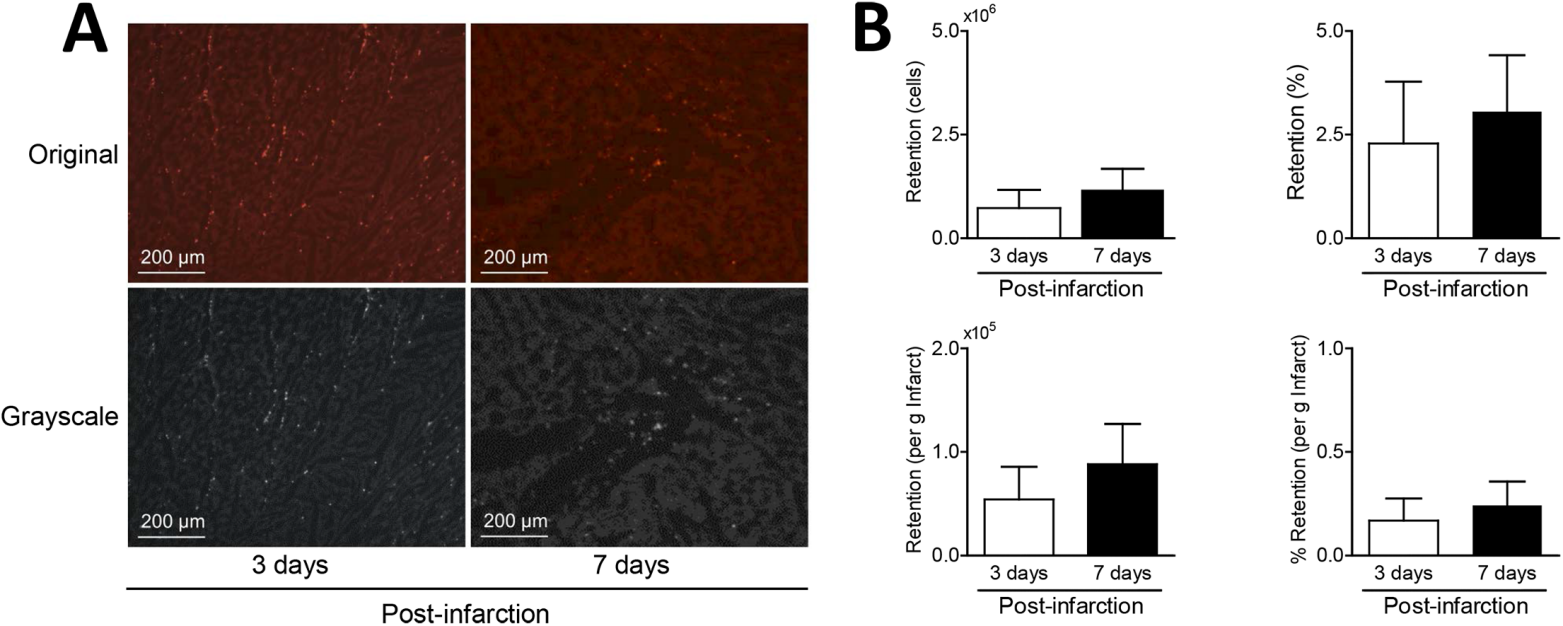
**Panel A**. Typical examples of BMMNC retention in infarcted myocardium at 3 days and 7 days post infarction. Presented are a selection of the original photos as well as the grayscale conversion used for further quantification. **Panel B.** Retention of cells in infarcted myocardium 3 days (n = 6) or 7 (n = 6) days post infarction expressed as absolute numbers or as a percentage of the initial dose and corrected for infarct mass (i.e. expressed per gram infarct). Data are expressed as mean ± SEM.

**Fig 3:**
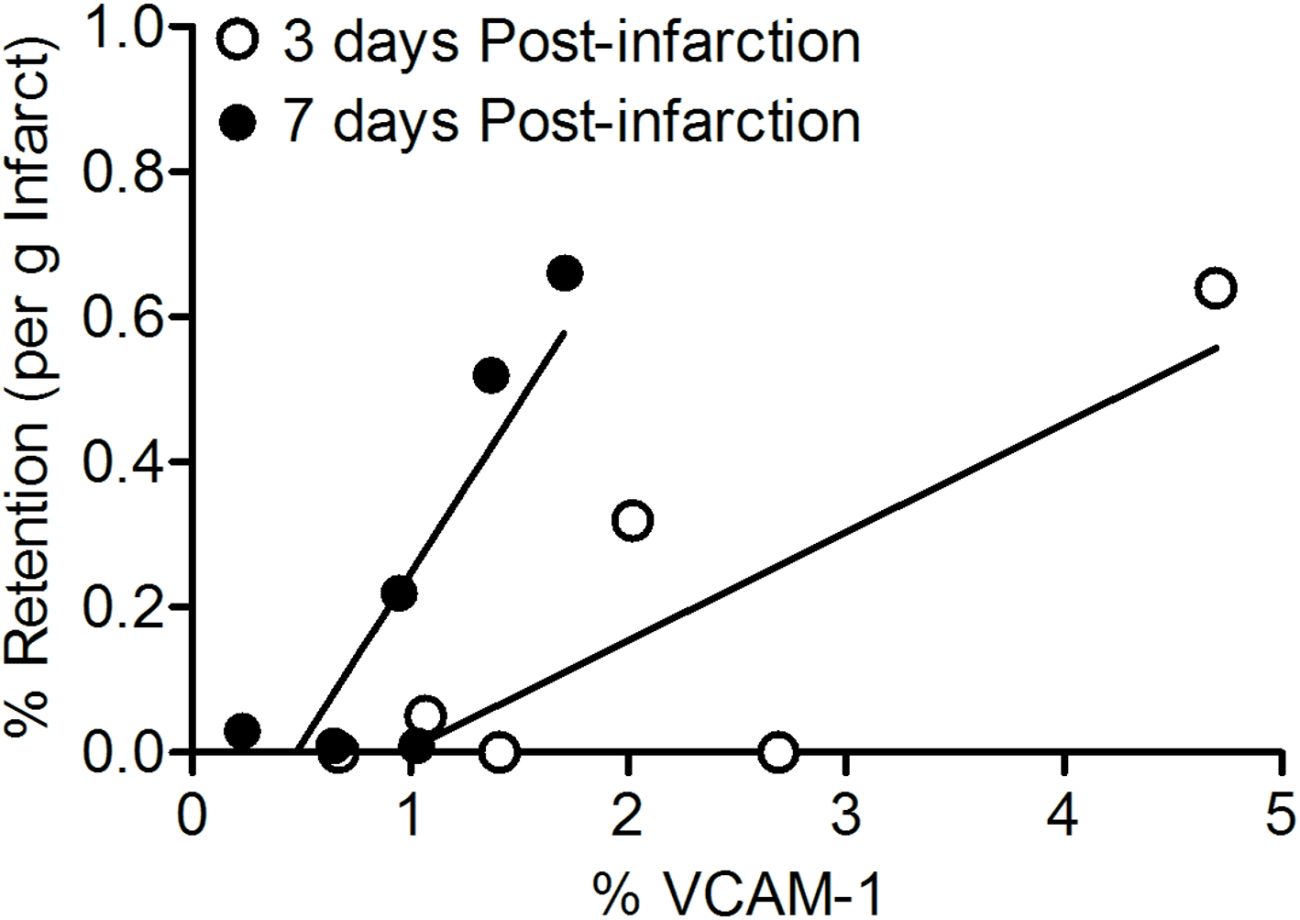
Regression analysis of VCAM-1 expression at 3 (○, r^2^ = 0.69, p = 0.03) and 7 days (●, r^2^ = 0.74, p = 0.04) days post-infarction vs. % of retained autologous bone marrow-derived cells per gram infarct.

**Fig 4:**
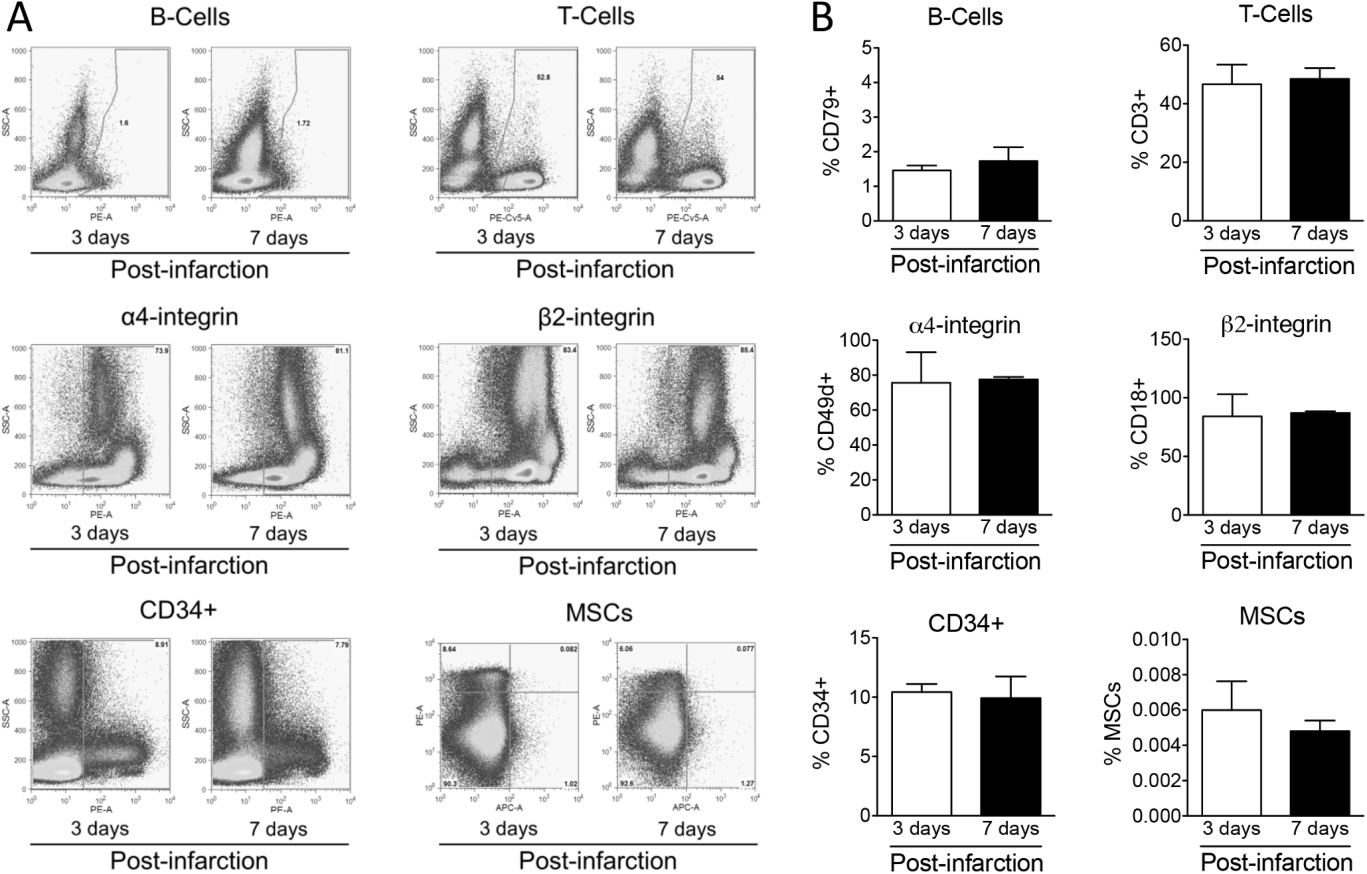
**Panel A**. Typical FACS flow plots of autologous bone marrow derived mononuclear composition studies. MSC = mesenchymal stem cell. **Panel B.** Composition of autologous infused bone marrow-derived mononuclear cells at 3 (n = 5) and 7 (n = 5) days post infarction. MSC = mesenchymal stem cell. Data are expressed as mean ± SEM.

Data are presented as mean ± SEM. Data in Fig 1 were analyzed with Sigmaplot (Version 11.0, Drunen, The Netherlands), using two-way (time x treatment) ANOVA followed by post-hoc Student-Newman-Keuls correction when appropriate. Data in Figs 2A and 4A were analyzed by unpaired t-test. Data in Fig 3 were analyzed using ANCOVA with % VCAM-1 as covariate and 3 and 7 days as independent factors. Statistical significance was accepted when p<0.05.

## Results

### VCAM-1 expression after acute myocardial infarction

#### Mortality and exclusion

Two out of 33 swine encountered non-convertible ventricular fibrillation during the ischemia-reperfusion protocol and could not complete the protocol. No premature euthanasia was performed as no animals suffered from human endpoint-associated (co)morbidities. Of the remaining 31 animals that successfully completed the protocol, 6 tissue-sets were ultimately not suitable for final analyses as a result of cryopreservation-induced tissue deformities leaving 25 analyzable datasets.

#### VCAM-1 presence after myocardial infarction

Fig 1A visualizes the transient presence of VCAM-1in remote and infarct tissue at 1, 3,7, 14 and 35 days post-AMI. Subsequent quantification of VCAM-1 (Fig 1B) shows that VCAM-1 presence in remote tissue remained low at all times (0.20±0.03%). In contrast, VCAM-1 presence in the infarct region was elevated at 3 days (2.41±0.62%, n = 4, p<0.001) and 7 days (0.98±0.28%, n = 5, p = 0.01) post-AMI. Importantly, VCAM-1 expression peaked at 3 days post-AMI and showed a transient pattern with normalization 14 days post-AMI.

#### VCAM-1 expression after myocardial infarction

Fig 1C confirms that VCAM-1 expression remained low at all times in remote tissue. VCAM-1 expression in infarct tissue homogenates confirm that VCAM-1 expression is less pronounced as compared to histological findings yet significantly upregulated in the sub-acute phase after reperfused myocardial infarction in a transient matter.

### Effects of VCAM-1 expression on cell retention

#### Mortality and infarct mass

Three out of 15 swine did not complete the protocol. Two swine that encountered ventricular fibrillation could not be converted to normal sinus rhythm and one swine died prematurely because of electromechanical dissociation during ischemia. No animals met the criteria for human endpoints or died after stratification into the 3 or 7 days post-AMI group or during cell infusion. Infarct mass at baseline, estimated from the plasma concentration of heart specific fatty acid binding protein determined at 50 min of reperfusion [24], was similar between the 3 day (11±2 g) and 7 day (13±4 g) groups (n = 6 per group; p = 0.65).

#### VCAM-1 presence

VCAM-1 presence in the cell retention studies was similar to the results of the first VCAM-1 histological studies (3 days post-AMI: 2.09±0.60 vs. 2.41±0.62%, p = 0.73; 7 days post-AMI: 0.99±0.21 vs. 0.98±0.28%, p = 0.97).

#### Cell retention

Initial 2-way ANOVA (time of administration x injection rate) analyses did not show any significant differences (P≥0.4). Consequently, fast and slow infusion results were pooled for further analyses and typical examples of immunofluorescence stainings in sections with BMMNC retention are shown in Fig 2A. Quantitative results are presented in Fig 2B and show that similar numbers of cells were infused in every group, 42±6·10^6^ cells at 3 days post-AMI vs. 43±4·10^6^ at 7 days post-AMI (p = 0.93). An average of 8.4±0.8 individual tissue samples were selected per animal and an average of 40±6 photos per animal were quantified for PKH26-positive cells. Results show that the absolute number of retained cells was not different at 3 or 7 days post-AMI (0.73·10^6^±0.44·10^6^ cells vs. 1.17·10^6^±0.51·10^6^, p = 0.52), with similar results when data were expressed as a percentage of the initially infused number of cells (2.3±1.3% vs. 3.1±1.4%, p = 0.72). Moreover, when results were corrected for infarct mass at baseline, results were again not statistically different for both absolute retention (0.054·10^6^±0.031·10^6^ cells/g vs. 0.088·10^6^±0.039·10^6^ cells/g, p = 0.51) as well as relative retention (0.17±0.11%/g vs. 0.24±0.12%/g, p = 0.65) at 3 and 7 days respectively.

#### VCAM-1 expression and cell retention

Post-AMI cell retention levels did not differ between 3 and 7 days although VCAM-1 expression levels were significantly higher at 3 compared to 7 days post-AMI. Interestingly, there were significant correlations between VCAM-1 expression and cell retention at both 3 days (r^2^ = 0.69, p = 0.03) and 7 days (r^2^ = 0.74, p = 0.04) post-AMI, so that higher expression of VCAM-1 was associated with a higher rate of cell retention within each group (Fig 3). However, the slope of the relation between VCAM-1 expression and retention tended to be lower at 3 as compared to 7 days post-AMI (p = 0.062). These findings suggest that while VCAM-1 is a determinant of cell retention, other factors must also play a role. One such factor could be the cell-composition of the mononuclear cell fraction that was harvested and injected at 3 days vs 7 days post-AMI.

### Effects of myocardial infarction on composition of the mononuclear fraction

Fig 4A and 4B show that the contribution of B-cells, T-cells, CD34+ cells and MSCs to the mononuclear cell fraction was similar at both time points. Furthermore, cell surface adhesion molecules α4 (part of VCAM-1) integrin and β2 (part of ICAM-1) integrin showed similar expression levels in both groups. Thus, no differences in composition of the infused fraction were observed between 3 and 7 days post-AMI. Similarly, there were no significant correlations noted between composition of the injected cells and magnitude of cell retention per gram of infarcted tissue (n = 5 per group). For B-cells (day 3: r^2^ = 0.61, p = 0.12; day7: r^2^ = 0.00, p = 0.94), T-cells (day 3: r^2^ = 0.07, p = 0.67; day7: r^2^ = 0.04, p = 0.76), CD34+ cells (day 3: r^2^ = 0.04, p = 0.76; day7: r^2^ = 0.41, p = 0.25) or MSCs (day 3: r^2^ = 0.16, p = 0.50; day7: r^2^ = 0.66, p = 0.09).

## Discussion

The present study investigated the temporal presence of VCAM-1 in infarcted and remote myocardial regions in swine with reperfused AMI, and its correlation with retention of autologous bone marrow-derived mononuclear cells harvested and injected at 3 or 7 days post-AMI. The major findings were that: (*i*) vascular cell adhesion molecule 1 (VCAM-1) expression is upregulated in the microcirculation of infarct-impaired myocardial tissue in a transient manner with VCAM-1 presence peaking at 3 days (~12- fold) and 7 days (~5-fold) post-AMI, with normalization to baseline values within 14 days post-AMI; (*ii*) VCAM-1 expression correlated with the magnitude of cell retention both at 3 days and 7 days post-AMI, but average cell retention was not different at 3 days vs. 7 days, indicating that cell retention was primarily independent of VCAM-1 presence; (*iii*) composition of the mononuclear fraction was not different 3 or 7 days post-AMI and selected cell types individually did not correlate with retention. The implications of these findings will be discussed.

### Cell retention after cell therapy

The limited therapeutic efficacy of cell therapy reported in clinical studies [1, 2] could, at least in part, be due to the relatively low retention rates of administered cells. Retention rates as high as 57.7% of the infused fraction [11] or as low as 0.8% of the infused fraction [13] have been reported after intracoronary infusion (Table 1). Also, no uniform approach in retention studies exists owing to the variations in parameters that can influence retention. These differences include, but are not limited to, the number and types of cells infused, as well as the timing and procedure of administration including infusion density and speed. Importantly, the preferred tracking method appears to be scintigraphy as it was used in ~83% of retentions studies. This method however, in which cells are labeled with a radioactive tracer does not correct for the heterogeneous label efficiency that exists when a heterogeneous cell population including differences in cell type and cell size is taken into account. This may lead to skewed results when a subpopulation of large cells containing much radiolabel is retained primarily [28]. Also radiolabeled cell debris may result in false-positive retention. Here we assessed BMMNC retention in detail using a histopathological approach of the complete infarct region This detailed approach, not affected by false positive scoring or differences in heterogeneous label efficiency, may explain our relatively low retention rates compared to other studies.

**Table 1:** Studies on intracoronary cell retention for cardiac cell therapy.

Author	Species	# Cells injected (1·10^6^)	Cell type	Cell Size	Infusion parameters	Enrichment protocol	Tracking method	Timing of injection post MI	FU after injection	Cardiac Retention (%)	Cardiac Retention Absolute
Hofmann et al.[10]	Human	2540	BMC	-	4–5 injections	gelatine-polysuccinate + immunomagnetics	Scintigraphy	5–10 days	50–75 min	2.1±0.4	5.3·10^7^
(2005)		24	CD34+							25.7±7.3	6.2·10^6^
Penicka et al.[18]	Human	2740	BMC	-	24ml total	-	Scintigraphy	9 days	2h	5	13.7·10^7^
(2005)					4.5-5ml injections				18h	1	27.4·10^6^
Blocklet et al.[5]	Human	15	PBCD34^+^	-	2-3x2ml	Cytapheresis + immunomagnetics	Scintigraphy	7–21 days	1 h	5.5±2.3	0.8·10^6^
(2005)											
Goussetis et al.[9]	Human	16	CD133^+^ +	-	5min/2·10^6^/min	Ficoll + immunomagnetics	Scintigraphy	45±36 months	1 h	9.2±3.6	1.5·10^6^
(2007)			CD133^-^CD34^+^						24 h	6.8±2.4	1.1·10^6^
Dedobbeleer et al.[6]	Human	18	CD34^+^	-	3x2ml	Cytapheresis + immunomagnetics	Scintigraphy	20±2 months	1 h	3.2±0.6	0.6·10^6^
(2009)											
Silva et al.[37]	Human	100	BMMNC	-	10ml/3x2-3min	Ficoll	Scintigraphy	5.5±1.3 days	4h	16.1±7.1	16.4·10^6^
(2009)					~10·10^6^ per min				24h	10.3±6.4	10.3·10^6^
Musialek et al.[16]	Human	4.2	CD34+	-	3x3.3ml in 3x3min	Ficoll + immunomagnetics	Scintigraphy	6–14 days	1h	4.9±0.5	0.21·10^6^
(2010)		4.5			3x10ml bolus					5.1±0.5	0.23·10^6^
Moreira et al.[15]	Human	100	BMMNC	-	10ml/3x2-3min	Ficoll	Scintigraphy	5.5±1.3 days	4h	16.1	16.4·10^6^
(2011)					~10·10^6^ per min				24h	10.3	10.3·10^6^
Musialek et al.[17]	Human	4.3	CD34+	-	30ml	Ficoll + immunomagnetics	Scintigraphy	5–10 days	1 h	5.2	0.22·10^6^
(2012)											
Hou et al.[12]	Pig	10	hPBMNC	-	30–45 sec	Ficoll	Scintigraphy	5–7 days	1h	2.6±0.3	0.3·10^6^
(2005)											
Freyman et al.[8]	Pig	50	Allogenic MSC	10–20^2^m	14ml/7x2 min	Density gradient centrifugation	Scintigraphy	15 min	14±3 days	6	2.9±1.0·10^6^
(2006)					3.5·10^6^ per min						
Moelker et al.[14]	Pig	25	BMMNC	5–7^2^m	5ml/5min	Lymphoprep	Histology	7 days	4 days	6.5	1.6·10^6^
(2006)					5·10^6^ per min						
Doyle et al.[7]	Pig	30	CPC	-	12ml/3x4ml/2.5·10^6^/ml	Ficoll +Expansion	Scintigraphy	2 days	1 h	8.7±1.5	2.6·10^6^
(2007)					30·10^6^ cells in 4ml/					17.8±7.9	5.3·10^6^
					2min bolus/15·10^6^/min						
Tossios et al.[19]	Pig	100	BMMNC	-	20ml/4x1min	Ficoll	Scintigraphy	5 days	1h	4.1±1.1	4.1·10^6^
(2008)					25·10^6^ per min				24h	3.0±0.6	3.0·10^6^
Ly et al.[13]	Pig	20	MSC	-	5ml in 3min	Ex-vivo expansion Histopaque Histopaque	NIR	3–4 days	Immediately	1.3±0.8	0.26·10^6^
(2009)			BMMNC		6.7·10^6^ per min					0.8±0.1	0.16·10^6^
			PBMNC							0.8±0.1	0.16·10^6^
Hong et al.[38]	Pig	10	Allogenic ASC	-	10ml/3x3min	-	Scintigraphy	6 days	1h	57.2±12.7	5.7±1.0·10^6^
(2014)					1.1·10^6^ per min				24h	22.6±5.5	2.3±1.0·10^6^
Keith et al.[20]	Pig	10	hCSCs	-	Flow + bolus	Ex-vivo expansion + Immummagnetics + Ex-vivo expansion	Scintigraphy	1–2 months	24h	5.4±0.8	0.5±1.0·10^6^
(2015)					Stop-flow + bolus					4.9±0.6	0.5±1.0·10^6^
Uitterdijk et al.	Pig	43	BMMNC	6–12^2^m	1·10^6^ per min	Lymphoprep	Histology	3 days	1h	2.3±1.5	0.7±0.4·10^6^
(2017)					5·10^6^ per min			7 days		3.1±1.4	1.2±0.5·10^6^

MI = myocardial infarction; FU = follow-up; BMC = bone marrow cells; CD = cluster of differentiation; PB = peripheral blood-derived; BMMNC = bone marrow-derived mononuclear cells; hPBMNC = human peripheral blood-derived mononuclear cells; MSC = mesenchymal stem cell; CPC = cardiac progenitor cell; ASC = adipose-derived stem cells; hCSCs = human cardiac stem cells.

### Post-infarct endothelial response

Upon AMI, endothelium within the affected area is activated resulting in a proinflammatory and procoagulant environment characterized by increased interactions with leukocytes [29]. Numerous adhesion factors are upregulated within the affected area including VCAM-1 [30]. VCAM-1 is expressed after infarct-induced cytokine release and serves as a “docking station” for leukocytes to facilitate the regional immune response [31]. Understanding the post-AMI up-regulation pattern of regional VCAM-1 may reveal the optimal timing for intracoronary infused bone marrow-derived mononuclear cell therapy. Here, we report the temporal expression pattern of VCAM-1 in the microcirculation of porcine ischemia-reperfusion impaired myocardium. VCAM-1 expression peaked at 3 days post-AMI and was normalized after 14 days, suggesting that cell retention would be optimal when applied at a time point that VCAM-1 expression is highest. Results of qPCR experiments confirm a significant and transient upregulation of regional VCAM-1 in the infarct zone in a transient matter in the sub-acute phase after AMI but do not fully corroborate with histological findings. We hypothesize that VCAM-1 here may be partially non-differentially expressed, differences in half-life of the VCAM-1 protein and mRNA may be different or other not-sufficiently defined post-transcriptional mechanisms underlie discrepancies between our expression studies and histological findings [32]. Retention of autologous BMMNCs, however, when administered at time points in which VCAM-1 was significantly upregulated, only partly supported our hypothesis. Thus, while a correlation between increased VCAM-1 expression and cell retention was observed both at 3 days and 7 days post-AMI, average cell retention at 3 days vs 7 days was similar despite different levels of VCAM-1 expression. These results suggest that cell retention may not only be determined by total VCAM-1 expression, and could have been influenced by other factors, including (*i*) different composition of the injected BMMNCs at 3 vs 7 days post-AMI, and (*ii*) simultaneous changes in endothelial expression levels of other adhesion factors such as E-selectin [33], P-selectin [34] or ICAM-1 [35] from 3 to 7 days post-infarction, thereby masking an effect of the increased VCAM-1 expression at 3 compared to 7 days. Since the latter were difficult to study due to lack of commercially available specific and reliable antibodies for pigs, we investigated the former and studied whether a change in composition of the injected BMMNCs at 3 and 7 days could play a role. The results showed that the composition was not different between 3 and 7 days. Clearly, future studies are required to further investigate the role of VCAM-1 in BMNNC retention in more detail, by determining the influence of other adhesion molecules (which the lack thereof is a limitation of the present study), in post-AMI cell-retention and study which cell types within the BMMNC injectate are primarily retained within the infarcted myocardium. Finally, the observations in the present study in swine may also provide an explanation for the results of the TIME-trial [36], that showed that treating patients with intracoronary autologous bone marrow-derived cells at 3 days, as compared to 7 days, post-AMI showed no significant improvement in the recovery of global or regional left ventricular function at six months follow-up.

### Composition of mononuclear cell fraction

Analysis of the BMMNC fraction isolated at 3 days or 7 days post-AMI did not reveal any differences in composition. In addition, the nature of the quantification study and the scarce availability of porcine antibodies restricted the phenotypical identification of retained cells within the infarct area and is considered a major limitation of this work. For future optimization studies, it remains of great interest to determine which cell type is dominant in retention studies. Our results however, do enable us to exclude that retained cells are MSCs only, as the average absolute number of MSCs is limited to ~2000–2500 cells per infused fraction whereas absolute retained number of cells approximate at least 0.7·10^6^ cells. Thus, cell composition of the BMMNC isolated 3 or 7 days post-AMI did not have an effect on absolute or relative cell retention suggesting that the role of composition of the cell infusate may not be decisive.

## Conclusions

The present study in swine with a reperfused AMI demonstrates that VCAM-1 is significantly upregulated in the microvasculature of infarcted myocardial tissue in a transient manner peaking at 3 days post-AMI with normalization to baseline at 14 days post-AMI. Although VCAM-1 expression correlated with the magnitude of cell retention at either 3 or 7 days post-AMI, the absolute and relative retention rates of BMMNCs were similar between these time points. This was not due to differences in the composition of infused bone marrow cell fractions, as cell composition of the infused BMMNC fractions was similar at 3 and 7 days post-AMI. Taken together, these findings indicate that VCAM-1 expression is not the principal determinant of BMMNC retention in reperfused myocardium post-AMI.

## Supporting information

S1 Fig Typical example of PKH26 label efficiency in BMMNC-retention studies. Results of typical PKH26 staining confirm >99% staining efficiency and differences in fluorescent avidity.
(TIF)
