# Supplementary figures and images for: Time course of VCAM-1 expression in reperfused myocardial infarction in swine and its relation to retention of intracoronary administered bone marrow-derived mononuclear cells
Source: PLoS One. 2017 Jun 19;12(6):e0178779. doi: 10.1371/journal.pone.0178779 (PMC5476248; doi:10.1371/journal.pone.0178779)

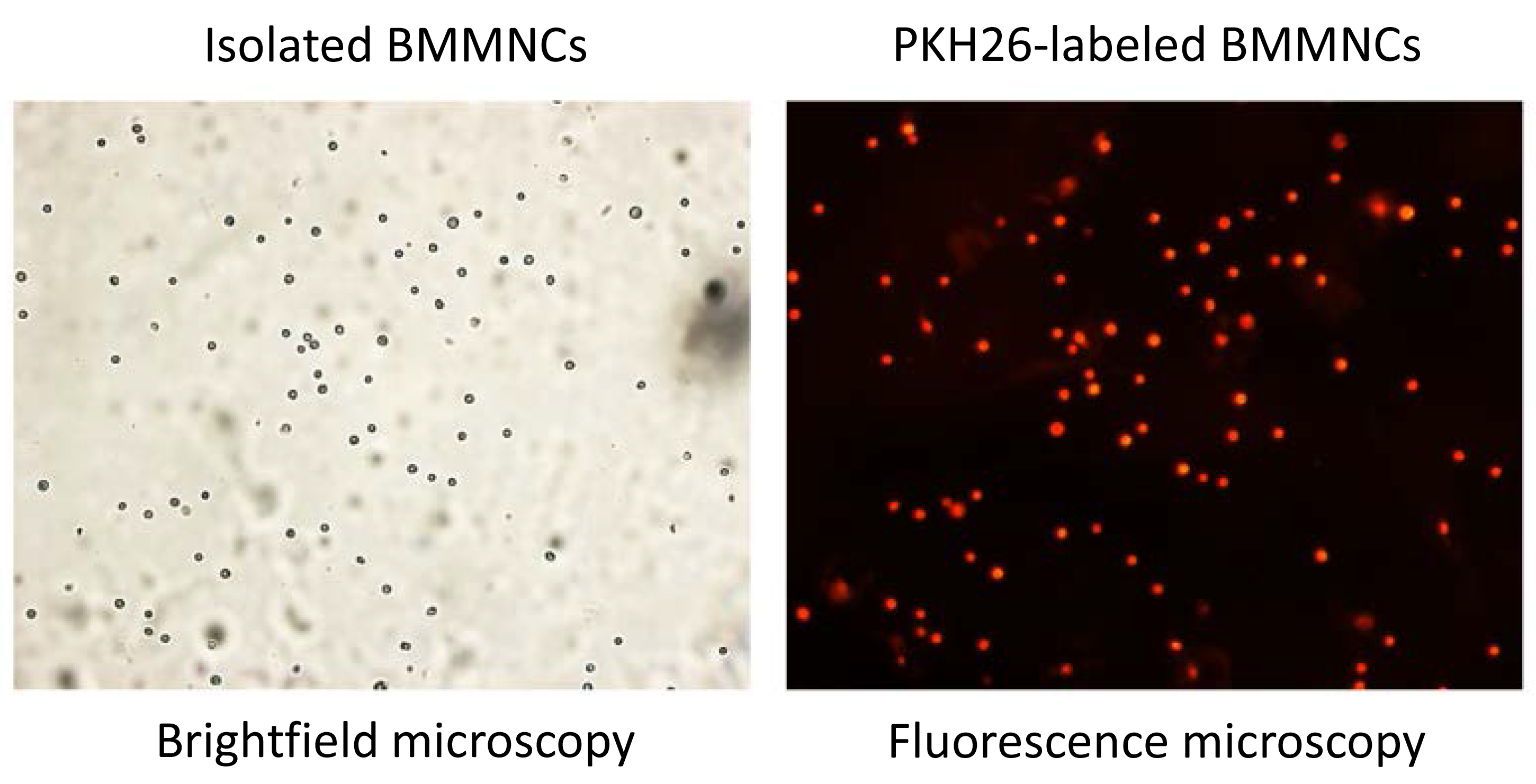

Supplement: S1 Fig — Results of typical PKH26 staining confirm >99% staining efficiency and differences in fluorescent avidity. (TIF) [file pone.0178779.s001.tif]
